# Reservoir computing with dielectric relaxation at an electrode–ionic liquid interface

**DOI:** 10.1038/s41598-022-10152-9

**Published:** 2022-04-28

**Authors:** Sang-Gyu Koh, Hisashi Shima, Yasuhisa Naitoh, Hiroyuki Akinaga, Kentaro Kinoshita

**Affiliations:** 1 Department of Applied Physics, Tokyo University of Science, 6-3-1 Niijuku, Katsushika, Tokyo 125-8585 Japan; 2 Device Technology Research Institute, National Institute of Advanced Industrial Science and Technology, Tsukuba Central 5, 1-1-1 Higashi, Tsukuba, Ibaraki 305-8565 Japan

**Keywords:** Electrical and electronic engineering, Electronic devices

## Abstract

A physical reservoir device with tunable transient dynamics is strongly required to process time-series data with various timescales generated in the edge region. In this study, we proposed using the dielectric relaxation at an electrode–ionic liquid (IL) interface as the physical reservoir by making the most of designable physicochemical properties of ILs. The transient dynamics of a Au/IL/Au reservoir device were characterized as a function of the alkyl chain length of cations in the IL (1-alkyl-3-methylimidazolium bis(trifluoromethane sulfonyl)imide). By considering a weighted sum of exponentials expressing a superposition of Debye-type relaxations, the transient dynamics were well reconstructed. Although such complex dynamics governed by multiple relaxation processes were observed, each extracted relaxation time scales with a power law as a function of IL’s viscosity determined by the alkyl chain length of cations. This indicates that the relaxation processes are characterized by bulk properties of the ILs that obey the widely received Vogel-Fulcher-Tammann law. We demonstrated that the 4-bit time-series signals were transformed into the 16 classifiable data, and the data transformation, which enables to achieve higher accuracy in an image classification task, can be easily optimized according to the features of the input signals by controlling the IL’s viscosity.

## Introduction

Edge artificial intelligence (AI) technology, which dispersively executes data processing in the peripheral domain of distributed ambient devices, is desirable for further implementing AI into our society^1^. Edge AI requires low-power and high-speed data processing^2,3^. These characteristics are indispensable, especially when processing environmentally generated time-series data in real time^4,5^. Under these circumstances, physical reservoir computing (PRC), which simplifies the computing paradigm of AI by utilizing the transient dynamics of physical systems, has recently attracted considerable attention for edge AI technology^6–10^. The PRC system is mainly composed of three layers: input, physical reservoir, and readout, as shown in Supplementary Fig. S1 online. The input time-series signals are transformed into easily classifiable spatiotemporal patterns through the physical reservoir that has fading memory and nonlinear properties. The physical reservoir plays the same role as the recurrent/coupling network with nonlinear nodes in conventional RC models, represented by the echo state networks or liquid state machines^11^. In the learning process, the weights on the connections are optimized only in the readout with a simple learning algorithm such that the data processing cost and computational time can be significantly reduced compared with conventional methods such as a recurrent neural network^12^. Until now, PRC based on various kinds of physical systems, such as volatile memristors^13,14^, quantum bits^15^, magnetic tunnel junctions^16^, spin torque oscillators^17^, mechanical oscillators^18^, opto-electronic architectures^19^, and processing-in-memory chip platforms^20^ has been proposed and demonstrated experimentally or computationally. This has successfully opened a paradigm of next-generation information processing powered by physical systems. However, the use of these physical reservoirs is still limited to applications with particular timescales because the controllability of their transient dynamics is not sufficient in terms of material engineering. It is important that the transient response attenuates with a relaxation time that is properly matched with the timescale of input signals to optimize the performance of the PRC. Therefore, a physical reservoir with tunable transient characteristics is required when extensive applications are assumed in the edge region.

To solve this problem, we focused on a dielectric relaxation at an electrode–ionic liquid (IL) interface. ILs are special salts that are in the liquid phase over a wide temperature range, typically from below room temperature to above 200 °C, and they have many attractive characteristics, including high ionic conductivity, negligible vapor pressure, non-flammability, and stability against external stimuli such as voltage, temperature, and pressure^21–23^. In addition, most ILs consist of organic ions; thus, their physicochemical properties are highly tunable by designing the molecular structure. Tokuda et al*.* reported that the viscosity of IL (1-alkyl-3-methylimidazolium bis(trifluoromethane sulfonyl)imide), which directly affects the ease of ion migration, can be systematically controlled by varying the alkyl chain length of cations^24^. Furthermore, it has been reported that the ILs can be handled as a solid electrolyte for device applications by loading the ILs into nanoporous materials or by incorporating the ILs into polymer gels, while keeping their superior characteristics^25,26^. Supplementary Fig. S2 online shows a schematic of the electrical double layers (EDLs) formed at the interface between the ILs and electrodes by applying a voltage. An ion rearrangement occurs to cancel out an external electric field shortly after the application of a voltage. As the cations and anions can move independently within ILs, the electric field is screened out completely from the bulk IL, and thereby confined in an extremely thin region on the electrodes. The region is reported to be nm scale and to be tuned by the surface potential and the IL ion structure^27,28^. This produces an enormous capacitance that cannot be achieved with conventional dielectrics such as SiO_2_^29,30^.

In this study, we proposed a physical reservoir device with tunable transient dynamics brought about by the dielectric relaxation at the electrode–IL interface. The transient current response of dielectric relaxation to input time-series signals was characterized by 2-terminal electrical measurement with a series of ILs, and a key parameter related to the transient characteristics was determined. Finally, an image classification task, in which the image data was converted into the pseudo time-series signals, was demonstrated using the proposed reservoir device to simply assess how varying the transient dynamics influences the classification accuracy.

## Results and discussion

### Characterization for transient response of dielectric relaxation at electrode–ionic liquid interface

**Figure 1:**
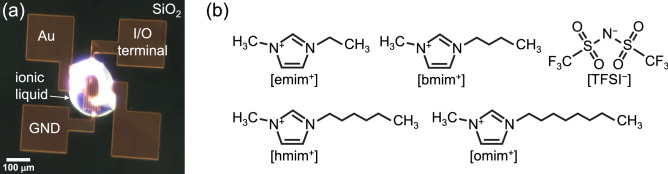
(**a**) Optical micrograph of Au/IL/Au reservoir device fabricated by dropping ILs onto Au gap electrode patterned on the SiO_2_ surface layer of Si substrate. (**b**) Structural formulas of emim^+^, bmim^+^, hmim^+^, and omim^+^ cations (left-hand side) and TFSI^−^ anion (right-hand side) in [Rmim^+^][TFSI^−^].

**Figure 2:**
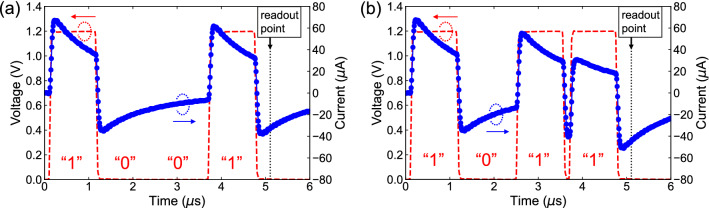
Dynamic current responses of Au/[bmim^+^][TFSI^−^]/Au to input voltage pulse trains with encoded 4-bit patterns, corresponding to (**a**) “1001” and (**b**) “1011.” Red dashed lines indicate applied voltage and blue circles indicate measured current. Output current (*I*_out_) is defined as current that is measured at readout point of 300 ns after applying the 4-bit pulse trains.

We fabricated Au/IL/Au reservoir devices by dropping ILs onto Au gap electrode patterned on the SiO_2_ surface layer of Si substrate, as shown in Fig. 1a. A series of ILs, which are composed of a fixed cationic backbone and anionic structure with various alkyl chain lengths, that is, 1-alkyl-3-methylimidazolium bis(trifluoromethane sulfonyl)imide ([Rmim^+^][TFSI^−^], R = ethyl (e), butyl (b), hexyl (h), and octyl (o)), were used in this study. The structural formulas of the Rmim^+^ cations, of which the alkyl chain length increases in the order of [emim^+^] < [bmim^+^] < [hmim^+^] < [omim^+^], and TFSI^−^ anion are shown in Fig. 1b. The Au/[emim^+^][TFSI^−^]/Au, Au/[bmim^+^][TFSI^−^]/Au, Au/[hmim^+^][TFSI^−^]/Au, and Au/[omim^+^][TFSI^−^]/Au reservoir devices were defined as emim_device—omim_device, respectively. The devices were characterized by 2-terminal electrical measurements where a bias voltage was applied to one of two electrodes whereas the other electrode was grounded. Figure 2a and b show two measured dynamical current responses of the bmim_device to input voltage pulse trains with encoded 4-bit patterns, corresponding to “1001” and “1011,” respectively. The pulse amplitude, pulse width, interval time between pulses, and leading/trailing edge times were 1.2 V, 1 µs, 100 ns, and 50/50 ns, respectively. We presumed that there was no oxidation or reduction of ILs used in this study and no electrolysis of adsorbed water under the bias voltage of 1.2 V. This is because the voltage applied to the interface is within the electrochemical window of the ILs and water, even though in an extreme situation where all the 1.2 V is applied to one side of interfaces only^31,32^. It was observed that the current decayed non-linearly with time during the duration of the voltage pulse^33,34^, and it behaved in the same manner with a negative sign inverted when the voltage was changed to 0 V. The input square-wave pulse is composed of the Fourier components below 5 MHz, and thereby we assume that the electrode polarization, which corresponds to the charging and discharging processes of the EDLs formed at the interface between [bmim^+^][TFSI^−^] and Au, is dominant relaxation mode in the observed dielectric relaxation^35,36^. Here, we define the current that was measured at a readout point of 300 ns after applying the 4-bit pulse trains as *I*_out_. The *I*_out_ corresponding to the input patterns of “1001” and “1011” indicated different current values, − 32.8 µA and − 44.5 µA, respectively, although both of the 4th pulses were “1,” meaning that the *I*_out_ contained the time-series information on whether the 3rd applied pulses were “0” or “1.” The dynamic current responses to all the combinations of 4-bit pulse patterns for emim_device—omim_device are also shown in Supplementary Fig. S3a–d online, respectively.

Reservoir devices are required to always return a certain response to the same input signal^7^. We then assessed the endurance characteristics of bmim_device with respect to the repetitive voltage pulse application, as shown in Supplementary Fig. S4 online. It was indicated that there was no degradation for at least 10^6^ cycles, ensuring that the transient dynamics of the proposed reservoir device are inherently reversible and reproducible, and have no electrochemical reactions leading to corrosion of electrodes or decomposition of ILs. In addition, we confirmed that almost the same current responses can be obtained between five different bmim_devices by precisely controlling the amount of ILs when dropping onto the gap electrode, as shown in Supplementary Fig. S5 online.

**Figure 3:**
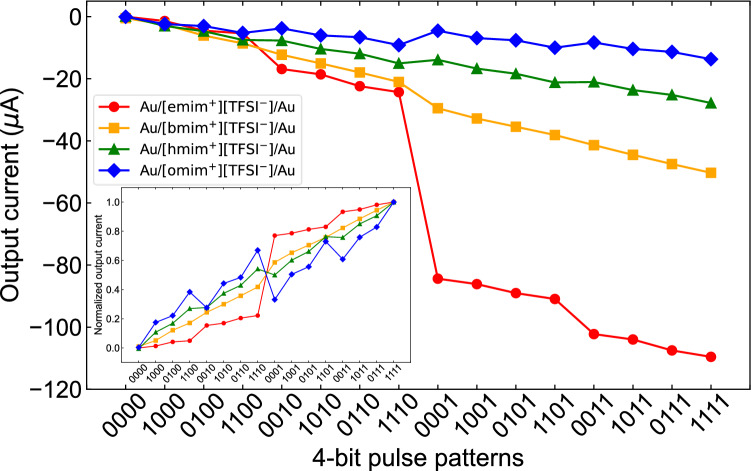
Output current (*I*_out_) extracted from dynamic current responses of Au/[emim^+^][TFSI^−^]/Au, Au/[bmim^+^][TFSI^−^]/Au, Au/[hmim^+^][TFSI^−^]/Au, and Au/[omim^+^][TFSI^−^]/Au, shown in Fig. S3a–d, as a function of 4-bit pulse patterns. The inset indicates *I*_out_ normalized with their maximum absolute value of *I*_out_ (< 0).

The *I*_out_ values extracted from the data in Supplementary Fig. S3a–d online are plotted in Fig. 3. It was confirmed that the extracted *I*_out_ values for each Rmim_divice were divided into 16 current values in a different manner as a function of 4-bit pulse patterns. Here, the order of 4-bit pulse patterns on the horizontal axis was arranged such that the absolute values of *I*_out_ (< 0) for emim_device and bmim_device were in ascending order. As the shorter alkyl chain length of cations leads to the lower viscosity of [Rmim^+^][TFSI^−^], which shows higher ionic conductivity, we observed that the shorter the alkyl chain length of cations in [Rmim^+^][TFSI^−^] was, the higher the absolute value of *I*_out_ measured with the pulse pattern of “1111” was. To make the increase and decrease in *I*_out_ as a function of 4-bit pulse patterns more visible, we normalized *I*_out_ for each Rmim_divice with the maximum absolute value of *I*_out_, as shown in the inset of Fig. 3. The normalized *I*_out_ for Rmim_device with a shorter alkyl chain length of cations increased primarily depending on the order of applied pulses rather than the number because a later pulse contributes more effectively to the increase in *I*_out_, owing to the faster relaxation of the transient response derived from the low viscosity. For example, the normalized *I*_out_ corresponding to “1110” was much lower than that corresponding to “0001” for emim_device and bmim_device. In contrast, the increase in the normalized *I*_out_ for Rmim_device with a longer alkyl chain length of cations primarily depends on the number of applied pulses because of the slower relaxation of the transient response derived from the high viscosity. For example, the normalized *I*_out_ corresponding to “1110” was much higher than that corresponding to “0001” for hmim_device and omim_device. Further, the normalized *I*_out_ values shown in the inset of Fig. 3 were rearranged in ascending order, to compare the dependence of normalized *I*_out_ on the pattern of 4-bit pulses for each Rmim_divice, as shown in Supplementary Fig. S6 online. The normalized *I*_out_ shows mostly linear dependency on the pattern of 4-bit pulses over all 16 patterns for bmim_device, hmim_device, and omim_device. In other words, the normalized *I*_out_ increases almost uniformly with changing the pattern of 4-bit pulses from “0000” through “1000,” “0100,” …, “0111,” to “1111.” In contrast, the one for emim_device was mainly divided into two regions depending on whether the 4th applied pulses were “0” or “1;” therefore, the margins to discern *I*_out_ were substantially restricted to approximately 40%. The pattern dependence of normalized *I*_out_ for emim_device has the smallest slope among all the emim_device—omim_device, except for the steep slope between 4-bit pulse patterns of “1110” and “0001.” Based on the above results, it is suggested that the proposed reservoir device, Au/[Rmim^+^][TFSI^−^]/Au, enables the transformation of the 4-bit time-series data into the 16 classifiable data by utilizing the transient dynamics brought about by the dielectric relaxation at the electrode–IL interface. The results of data transformation are determined by the relation between the input signal’s timescale and the relaxation time which can be tuned by controlling the viscosity of [Rmim^+^][TFSI^−^] as a function of the alkyl chain length of the cations.

### Relation between relaxation time of dielectric relaxation and viscosity of [Rmim^+^][TFSI^−^]

**Figure 4:**
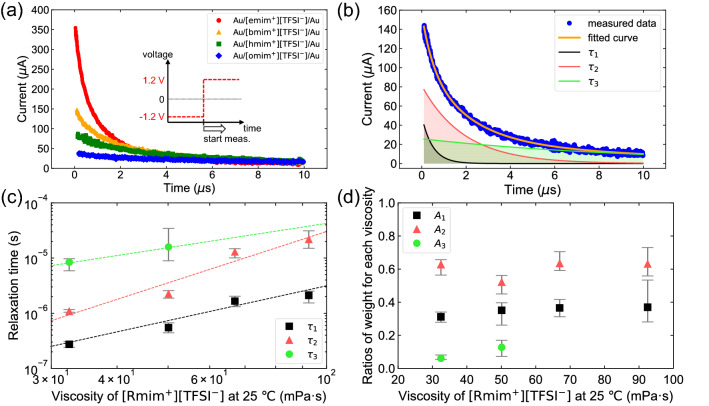
(**a**) Transient current responses measured with Au/[Rmim^+^][TFSI^−^]/Au as a function of alkyl chain length of cations in [Rmim^+^][TFSI^−^]. The inset indicates timing of current measurement during duration of voltage pulse. (**b**) Transient current response for Au/[bmim^+^][TFSI^−^]/Au with fitted curve simulated based on Eq. (1). Three types of Debye-type relaxations that constitute the fitted curve are shown by solid lines with shades. (**c**) Relaxation times as a function of the viscosity of [Rmim^+^][TFSI^−^]. Dashed lines represent results of fitting based on least-square method considering Eq. (2). (**d**) Ratios of weight for each viscosity as a function of viscosity of [Rmim^+^][TFSI^−^]. Plots for (**c**) and (**d**) indicate the average of 7 different devices and each error bar shows their minimum and maximum values.

Figure 4a shows the transient current responses of emim_device—omim_device which were measured under a bias voltage of 1.2 V for 10 µs. To ensure a measurable current, a bias voltage of − 1.2 V was applied until the measurement started at 1.2 V, as shown in the inset of Fig. 4a. The current decay became modest with an increase in the alkyl chain length of cations in [Rmim^+^][TFSI^−^], as well as the current decays observed in Supplementary Fig. S3a–d online. To extract the relaxation times ($\tau_{i}$ s), we fitted the transient current responses based on least-square method considering Eq. (1) which has a weighted sum of exponentials expressing a superposition of Debye-type relaxations^37,38^.

1 $$\begin{array}{*{20}c} {I_{transient} = \mathop \sum \limits_{i = 1}^{n} A_{i} \exp \left( { - \frac{t}{{\tau_{i} }}} \right)} \\ \end{array}$$

where $t$ and $A$_*i*_ indicate time and the weight of each Debye-type relaxation. As a reference, the fitted curve along with the transient current response for bmim_device is shown in Fig. 4b. The fitted curves for emim_device, hmim_device and omim_device, and the extracted parameters ($\tau_{i}$ s and $A$_*i*_s) are provided in Supplementary Fig. S7a–c and Table S1 online, respectively. The transient current responses were fitted well with Eq. (1), considering three types of relaxation processes for emim_device and bmim_device, and two types of relaxation processes for hmim_device and omim_device. Note that the third relaxation processes for hmim_device and omim_device are thought to be unmeasurable in the current measurement timescale. Figure 4c shows the extracted $\tau_{i}$ s as a function of viscosity ($\eta$) of [Rmim^+^][TFSI^−^] at 25 °C^39–42^. The plots indicate the average of $\tau$_*i*_ s obtained from 7 different devices and each error bar shows their minimum and maximum values. We observed that each $\tau$_*i*_ scales with a power law as a function of $\eta$, and the relation can be represented as follows,

2 $$\begin{array}{*{20}c} {\tau_{i} = B_{i} \eta^{{C_{i} }} \left( {\eta > 0} \right)} \\ \end{array}$$

where $B_{i}$ and $C_{i}$ are constants that may be affected by the device structure and/or relaxation mechanism. The relation represented by Eq. (2) can be derived by presuming that $\tau_{i}$ and $\eta$ follow Vogel-Fulcher-Tammann (VFT) relation, which is often used to describe bulk properties of ILs^43,44^,

3 $$\begin{array}{*{20}c} {\tau_{i} = \tau_{0i} exp\left( {\frac{{D_{i} }}{{T - T_{\tau i} }}} \right)} \\ \end{array}$$

4 $$\begin{array}{*{20}c} {\eta = \eta_{0} exp\left( {\frac{E}{{T - T_{\eta } }}} \right)} \\ \end{array}$$

where $T$ is absolute temperature and $\tau_{0i}$, $\eta_{0}$, $T_{\tau i}$, $T_{\eta }$, $D_{i}$ and $E$ are constants. Combining these VFT relations of Eq. (3) and Eq. (4), Eq. (2) is derived assuming that $T_{\tau i}$ is equal to $T_{\eta }$, as follows:

$$\tau_{i} = \frac{{\tau_{0i} }}{{\eta_{0}^{{\frac{{D_{i} }}{E}}} }}\eta^{{\frac{{D_{i} }}{E}}} \left( {\frac{{\tau_{0i} }}{{\eta_{0}^{{\frac{{D_{i} }}{E}}} }} = B_{i} {\text{ and }}\frac{{D_{i} }}{E} = C_{i} } \right).$$

Based on the above discussion, we concluded that there exist three different types of relaxation processes in the dielectric relaxation of Au/[Rmim^+^][TFSI^−^]/Au reservoir devices. Nevertheless, these relaxation processes are characterized by bulk properties of ILs that obey the widely received VFT law. In addition, the ratios of weight, $A_{i} /\left( {\mathop \sum \limits_{i = 1}^{n} A_{i} } \right)$ for each $\eta$, as a function of $\eta$ were almost invariant with varying $\eta$, as shown in Fig. 4d. This indicates that the contribution ratios of each relaxation mechanism, which gives each $\tau_{i}$, to the total current ($I_{transient}$) are almost the same regardless of $\eta$.

The ILs consist only of cations and anions without any solvent, and these features make ILs fundamentally different from the conventional electrolyte solution, especially at the electrode–IL interface. Using various techniques, such as X-ray reflectivity, frequency-modulation atomic force microscopy, and molecular dynamics simulation, several researchers have proved that the extremely high ion concentration of IL leads to the formation of a cation − anion layered structure at the electrode–IL interface^45–47^. Uysal et al*.* reported that the anions ([TFSI^−^]) may have several different orientations in the first adsorbed layer under a bias voltage with a graphene electrode, and it causes multiple sharp peaks in the electron density profile obtained by the X-ray reflectivity^48^. In addition, Perkin pointed out that the ion diffusion and structural relaxation within each layer involves different molecular interactions and energy barriers compared to diffusion between layers, based on the experiments for IL confined to thin films using a Surface Force Apparatus^49^. Based on these previous studies, the three types of relaxation processes observed in this study may be related to the complex cation–anion layered structure at the interface. At such an extraordinary interface, it is surprising that the relaxation time of the dielectric relaxation can be estimated by the parameter that characterizes the bulk properties of ILs, as indicated by Eq. (2). This suggests that the IL near the interface not only exhibits solid-like properties as already reported but also retains a correlation with the bulk IL. Meanwhile, it provides us a useful guideline for device engineering based only on bulk IL parameters, such as the viscosity as shown in the present work, without using information near the interfaces that are difficult to access. Further investigation is necessary because the origin of each relaxation process is still unclear.

### Demonstration of image classification task using proposed reservoir device

**Figure 5:**
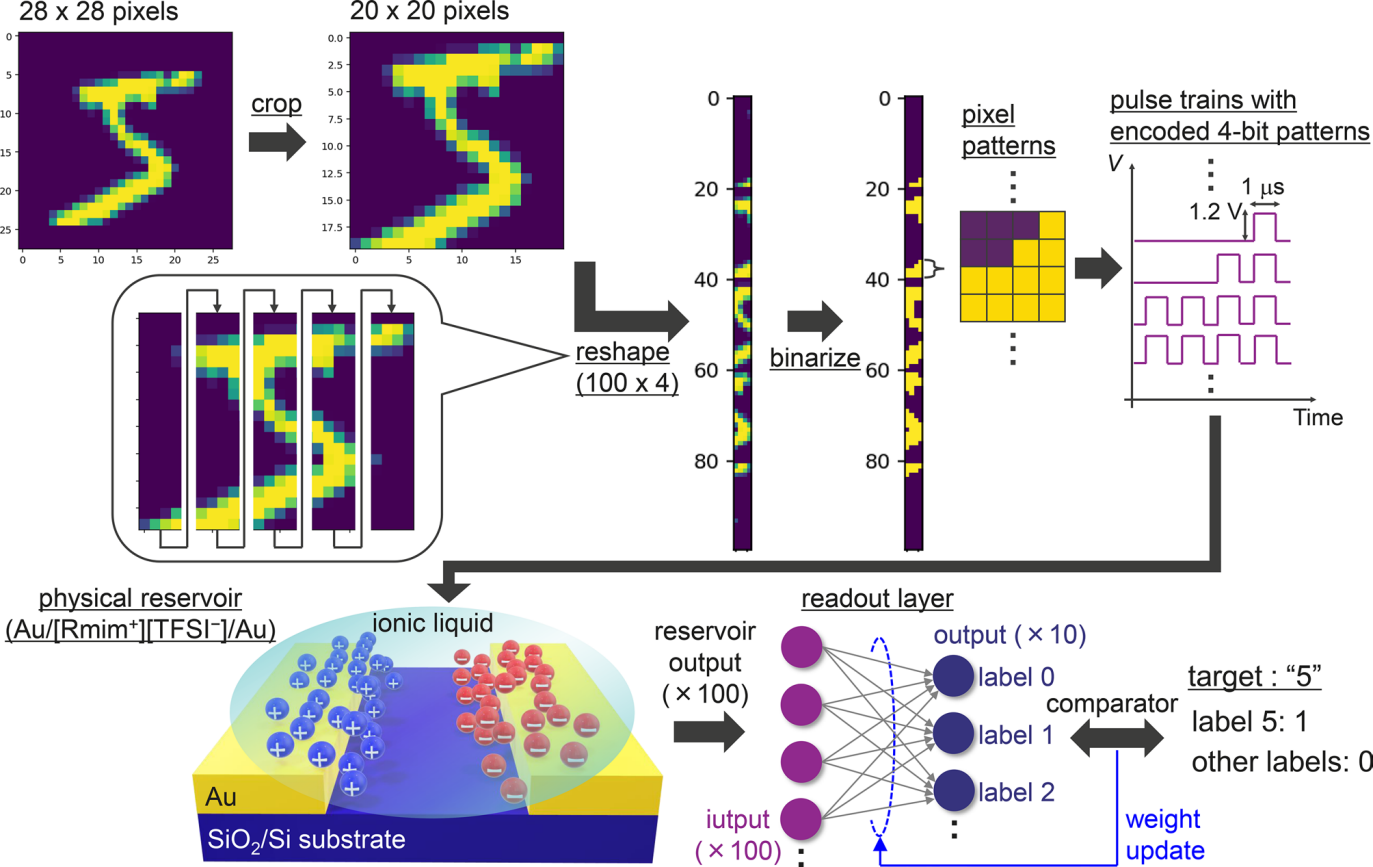
Schematic of the data-processing sequence for the image classification task in this study.

We then demonstrated an image classification task on the Modified National Institute of Standards and Technology (MNIST) dataset, where the image data were converted into pseudo time-series signals^13,14,50^, to simply assess how varying the transient dynamics influences the classification accuracy. Figure 5 shows a schematic of the data processing sequence for the image classification task in this study. The original 28 × 28-pixel images were cropped to 20 × 20 pixels to reduce the computational complexity by cutting down the part unrelated to the character. The images were then divided into five columns and sequentially joined with each other to shape 100 × 4-pixel images. Subsequently, the processed images were binarized and converted to pulse trains with encoded 4-bit patterns. The yellow and dark purple pixels were represented by a voltage pulse of 1.2 V and no voltage pulse (0 V), respectively. Each pulse train was then sent to the reservoir device and the *I*_out_ was recorded. The *I*_out_ was normalized with the maximum absolute value of *I*_out_ which corresponds to the input patterns of “1111”, and the vector of all 100 normalized *I*_out_ was used as the input to the readout layer, which is a single layer perceptron with 100 input nodes and 10 output nodes. Each output node corresponds to the label number from 0 to 9. Note that no device-to-device information mixing was included when obtaining the vector of 100 normalized *I*_out_. A supervised learning was executed in the readout layer, which had a softmax function in the output nodes, based on the error backpropagation that uses the RMSprop method to minimize the cross-entropy loss by updating each weight between the input and output. A similar approach was taken in many previous studies for the performance assessment of physical reservoir by the MNIST image classification task^13,14,50^. This process was repeated for 12 epochs using a batch size of 128 with 60,000 handwritten images from the MNIST training dataset, and it was then tested with 10,000 handwritten images from the MNIST test dataset. The entire learning process was executed 10 times, and the estimated classification accuracies were averaged to cancel the variability for each learning process.

**Figure 6:**
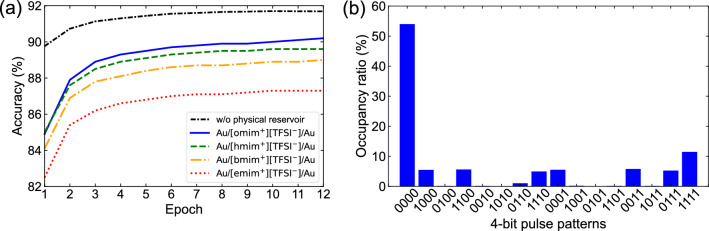
(**a**) Estimated accuracies of image classification using Au/[Rmim^+^][TFSI^−^]/Au until 12 epochs as a function of alkyl chain length of cations in [Rmim^+^][TFSI^−^]. Baseline performance of readout layer without physical reservoir is shown as comparison, where the number of weights which can be optimized increases by four times compared to when using the physical reservoir. (**b**) Occupancy ratios of each 4-bit pulse pattern for 6 $\times$ 10^6^ pulse trains (100 pulse trains $\times$ 60,000 training images) used in this study.

Figure 6a shows the estimated accuracies of the image classification using the emim_device—omim_device for 12 epochs as a function of the alkyl chain length of cations in [Rmim^+^][TFSI^−^]. It was confirmed that the accuracy improved with an increasing number of epochs, and 90.2%, 89.6%, 89.0%, and 87.3% accuracy was achieved at 12 epochs for omim_device, hmim_device, bmim_device, and emim_device, respectively. As expected from the dependence of the normalized *I*_out_ on the pattern of 4-bit pulses shown in Supplementary Fig. S6 online, the accuracies of omim_device, hmim_device, and bmim_device, whose normalized *I*_out_ almost linearly depends on the pattern of 4-bit pulses, was significantly higher than that of emim_device with the smallest slope of the dependence leading to smaller margins to discern the *I*_out_. Figure 6b shows the occupancy ratios of each pulse pattern in the 6 $\times$ 10^6^ pulse trains (100 pulse trains $\times$ 60,000 training images) used in this study. It was confirmed that the “0000” and “1111,” which correspond to the cases where four adjacent pixels are all blank and all filled, respectively, were dominant in the occupancy ratios of each pulse pattern. Accordingly, it is advantageous to use omim_device, whose *I*_out_ corresponding to the input patterns of “0000” and “1111” are distinctively divided from other *I*_out_ values, owing to the steep slopes of the pattern dependence of normalized *I*_out_ at both ends of Supplementary Fig. S6 online (“0000” and “1111” for omim_device). The order of achieved accuracies for other hmim_device and bmim_device can also be understood in the same way. These results indicate that the transient characteristics of the proposed reservoir device can be easily optimized according to the features of the input signals, and this leads to higher classification accuracy. For comparison, the baseline performance of readout layer was also estimated by directly sending the binarized 400 × 1-pixel images to the input of readout layer consisting of a single layer perceptron with 400 input nodes and 10 output nodes. It was indicated that the classification accuracy obtained with the physical reservoir was estimated to be lower than the baseline performance of readout layer. Here, the number of weights which can be optimized in the readout layer without physical reservoir increases by four times compared to when using the physical reservoir which plays a role to consolidate the 100 × 4-pixel data to 100 × 1 reservoir output. Therefore, this result can be understood by considering the fundamental concept of reservoir computing in the sense that the computing can be performed on low power owing to a small number of parameters (weighs) at the expense of some programmability^6^.

## Conclusions

We proposed the physical reservoir device, Au/[Rmim^+^][TFSI^−^]/Au, with tunable transient dynamics brought about by the dielectric relaxation at the electrode–IL interface. The characterization of the dielectric relaxation as a function of the alkyl chain length of cations for [Rmim^+^][TFSI^−^] revealed that the transient dynamics were well reconstructed by considering a weighted sum of exponentials expressing a superposition of Debye-type relaxations. Although such complex dynamics governed by multiple relaxation processes were observed, each extracted relaxation time scales with a power law as a function of IL’s viscosity which was determined by the alkyl chain length of cations. This indicates that the relaxation processes are characterized by bulk properties of the ILs that obey the widely received Vogel-Fulcher-Tammann law. The 4-bit time-series signals were transformed into the 16 classifiable data using the proposed reservoir device, and the data transformation can be easily optimized according to the features of the input signals by controlling the viscosity of [Rmim^+^][TFSI^−^]. This was suggested to be possible because the dielectric relaxation at the electrode–IL interface was corelated with parameters characterizing bulk physicochemical properties of the ILs. Finally, the image classification task on the MNIST dataset was demonstrated to simply assess how varying the transient dynamics influences the classification accuracy. The highest accuracy was achieved with omim_device because its transient dynamics attenuates with a relaxation time that is properly matched with the timescale of input signals used in the demonstration. The highly tunable transient dynamics brought about by the dielectric relaxation at the electrode–IL interface provide a suitable reservoir device for PRC systems assuming edge AI applications.

## Method

A Au film with a thickness of 60 nm was deposited on a 500-nm-thick thermally oxidized surface layer of a Si substrate by thermal evaporation through a shadow mask to construct planar gap electrodes. Subsequently, the ILs were dropped onto the gap electrode to form the Au/IL/Au reservoir device, as shown in Fig. 1a. Planar electrodes with dimensions of 200 µm-length × 10 µm-width × 60 nm-thickness, separated by a 10 mm-gap, were used in this study (see Supplementary Fig. S7 online). A series of ILs, 1-alkyl-3-methylimidazolium bis(trifluoromethane sulfonyl)imide ([Rmim^+^][TFSI^−^], R = ethyl (e), butyl (b), hexyl (h), and octyl (o)), were purchased from Tokyo Chemical Industry Co., Ltd. and used without further purification. The transient current response of dielectric relaxation to input time-series signals was characterized by 2-terminal electrical measurement using a semiconductor device analyzer with a waveform generator/fast measurement unit (Keysight Technologies, B1500A WGFMU). To extract an accurate relaxation time in the transient response, a setup involving a waveform generator (Keysight Technologies, 33522B) and a digital storage oscilloscope (Keysight Technologies, DSO-X 3054A) were also used. All the electrical measurements were conducted at around 24–25 °C in ambient air. To demonstrate the image classification task on handwritten images from the MNIST dataset, we used an open-source neural network library (Keras) for the supervised learning in the readout layer of our PRC system. A softmax function which is a kind of nonlinear functions was employed in output nodes of the readout layer. The use of such a nonlinear function requires the gradient-based learning such as the RMSprop method used in this study and can contributes the performance of the readout layer in exchange for increasing in some computational complexity. Although using purely linear output nodes would be sufficient to demonstrate the task^51^, we chose using the softmax function to enable comparison with the previous studies which took a similar approach for the performance assessment of physical reservoir by the MNIST image classification task^13,14,50^.

## Supplementary Information

Supplementary Information.

## Data Availability

The datasets generated during and/or analyzed during the current study are available from the corresponding author on reasonable request.
