# Supplementary material for: Reservoir computing with dielectric relaxation at an electrode–ionic liquid interface
Source: Sci Rep. 2022 Apr 28;12:6958. doi: 10.1038/s41598-022-10152-9 (PMC9051109; doi:10.1038/s41598-022-10152-9)
Supplement: Supplementary file 1 — Supplementary Information. [file 41598_2022_10152_MOESM1_ESM.pdf]

Supplementary Information

Reservoir computing with dielectric relaxation at an electrode–ionic liquid interface

Sang-Gyu Koh[1, 2], Hisashi Shima[2*], Yasuhisa Naitoh[2], Hiroyuki Akinaga[2], and Kentaro Kinoshita[1*]

[1]Department of Applied Physics, Tokyo University of Science, 6-3-1 Niijuku, Katsushika, Tokyo 125-8585, Japan

[2]Device Technology Research Institute, National Institute of Advanced Industrial Science and Technology, Tsukuba Central 5, 1-1-1 Higashi, Tsukuba, Ibaraki 305-8565, Japan

*E-mail: kkinosita@rs.tus.ac.jp, shima-hisashi@aist.go.jp

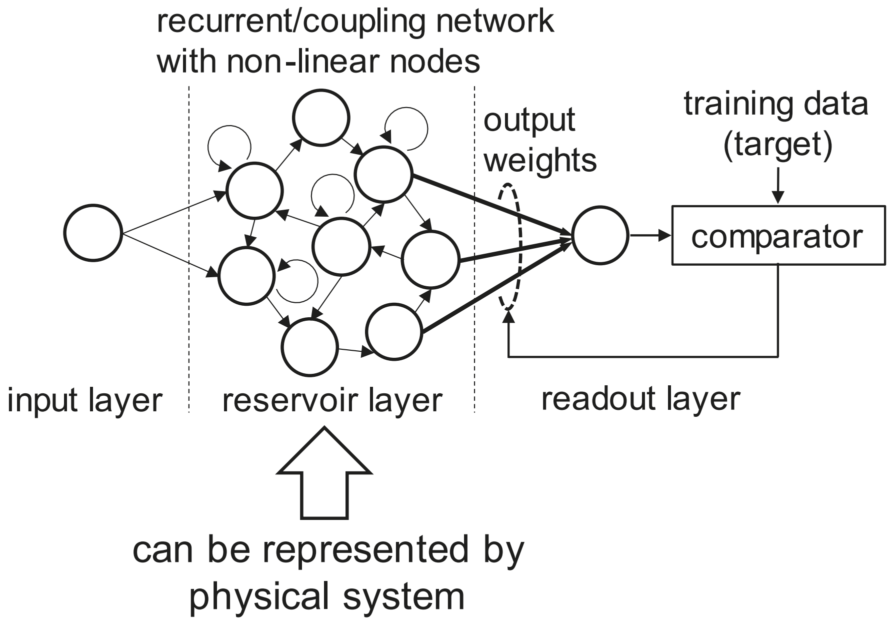

Figure S1. Schematic of physical reservoir computing system, which is mainly composed of 3 layers: input, physical reservoir, and readout. Input time-series signals are transformed into easily classifiable spatiotemporal patterns through the physical reservoir, which has fading memory and non-linear properties. The physical reservoir plays the same role as recurrent/coupling network with non-linear nodes in conventional reservoir computing models, represented by echo state networks or liquid state machines. In the learning process, weights on connections are optimized only in readout with a simple learning algorithm.

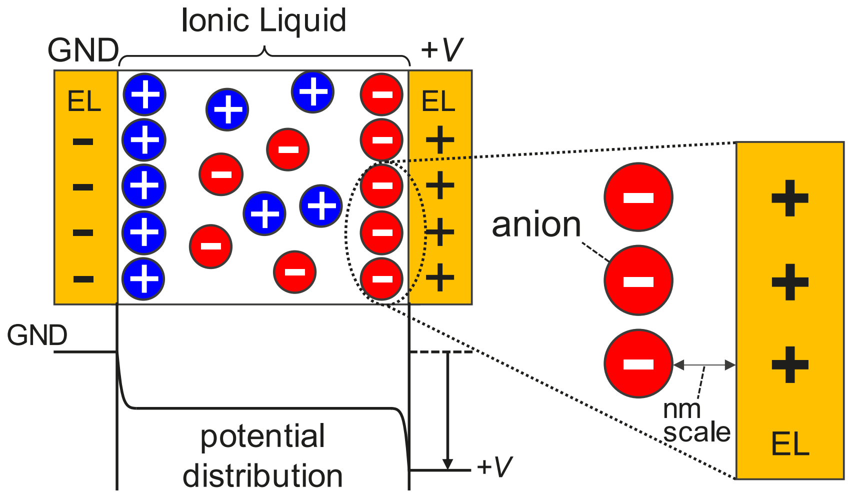

Figure S2. Schematic of electrical double layers formed at interface between ionic liquids and electrodes by applying voltage. Ion rearrangement occurs to cancel out external electric field shortly after application of voltage. As the cations and anions can move independently within ionic liquids, electric field is screened out completely from the bulk IL, and thereby confined in an extremely thin region (nm scale) on the electrodes.

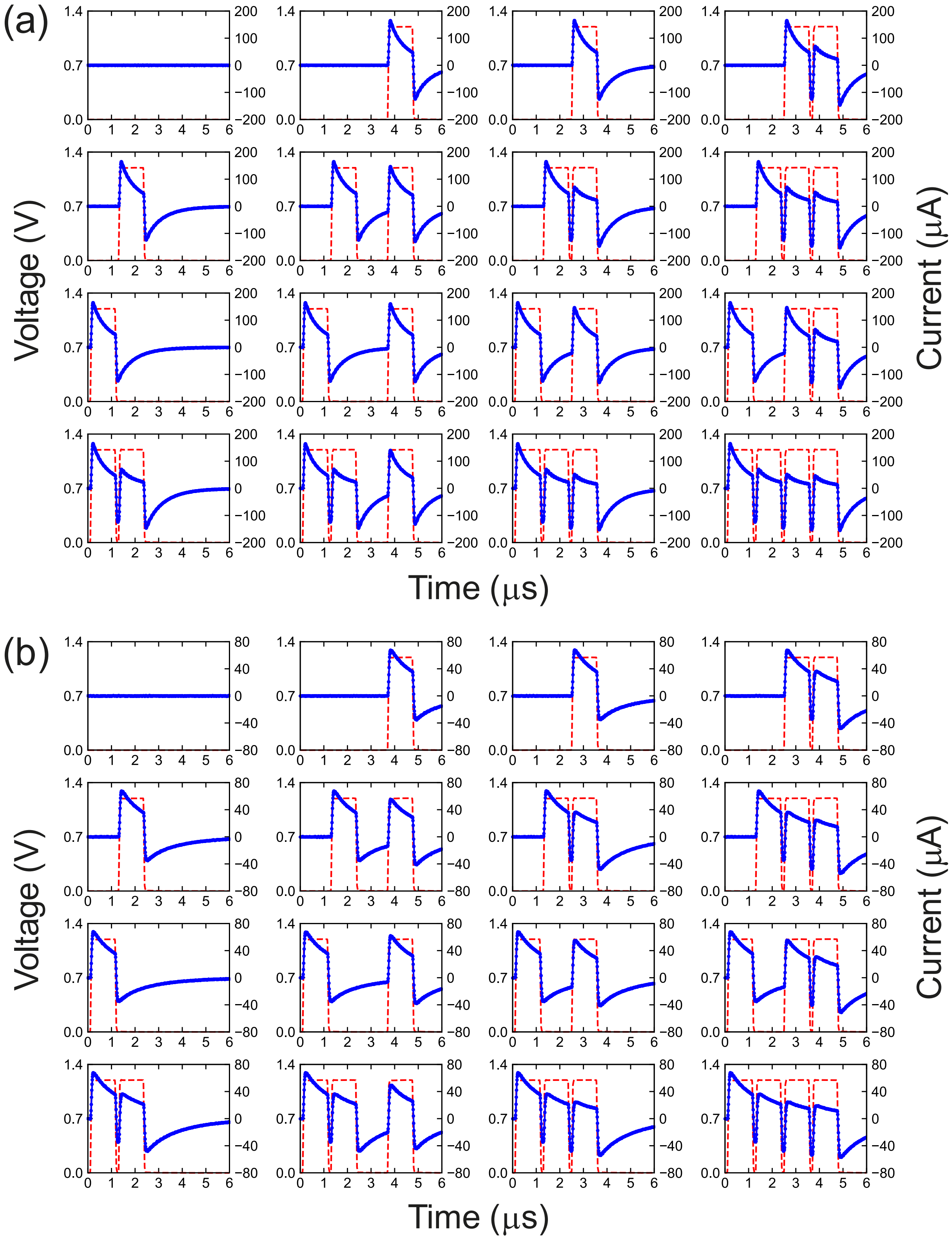

(a)
Voltage (V)
Current (μA)
Time (μs)
(b)
Voltage (V)
Current (μA)
Time (μs)

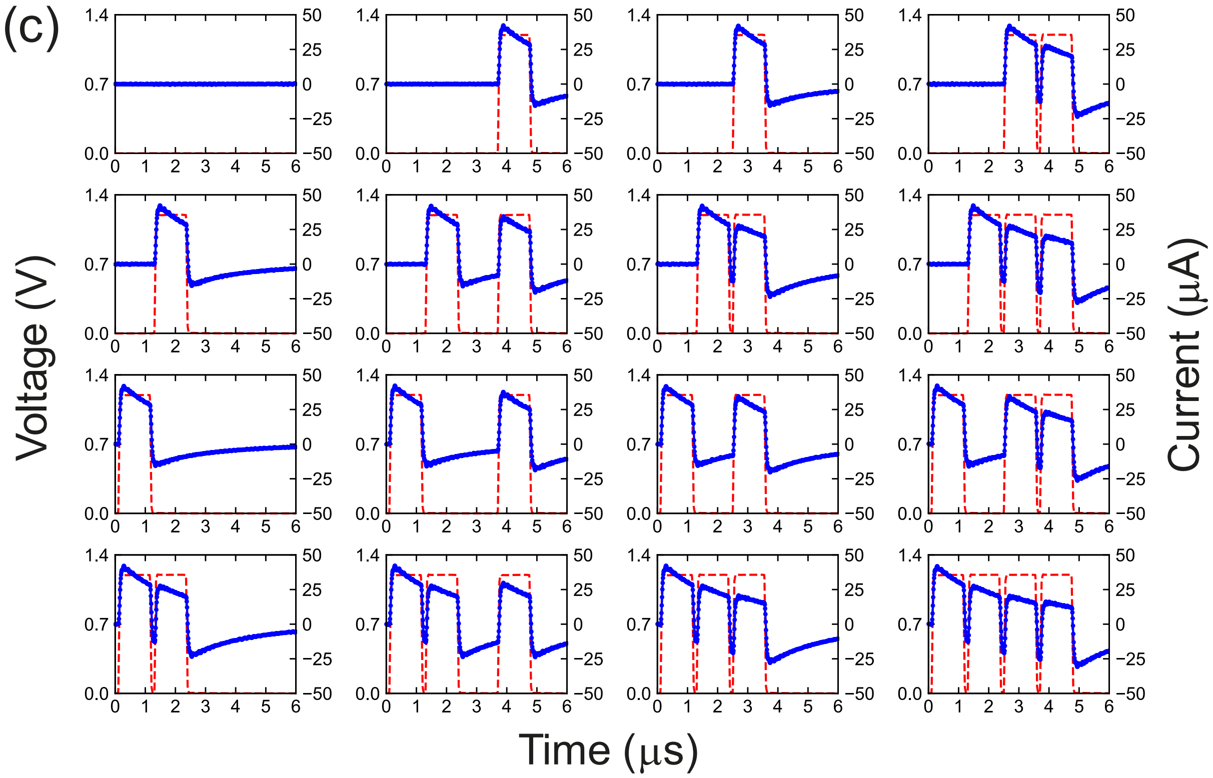

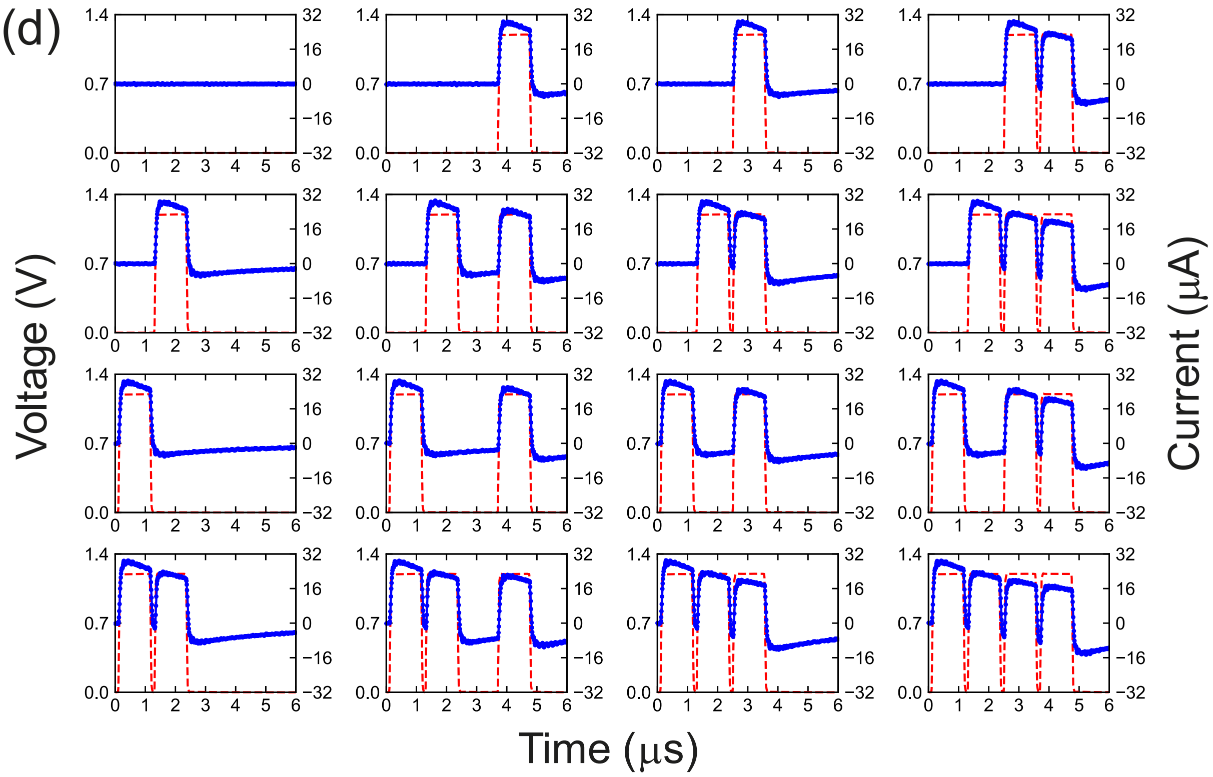

Figure S3. Dynamic current responses to all combinations of 4-bit pulse patterns from "0000" (upper left) to "1111" (bottom right) for (**a**) Au/[emim$^+$][TFSI$^-$]/Au, (**b**) Au/[bmim$^+$][TFSI$^-$]/Au, (**c**) Au/[hmim$^+$][TFSI$^-$]/Au, and (**d**) Au/[omim$^+$][TFSI$^-$]/Au. Dashed line indicates voltage and solid line with circle plots indicates measured current.

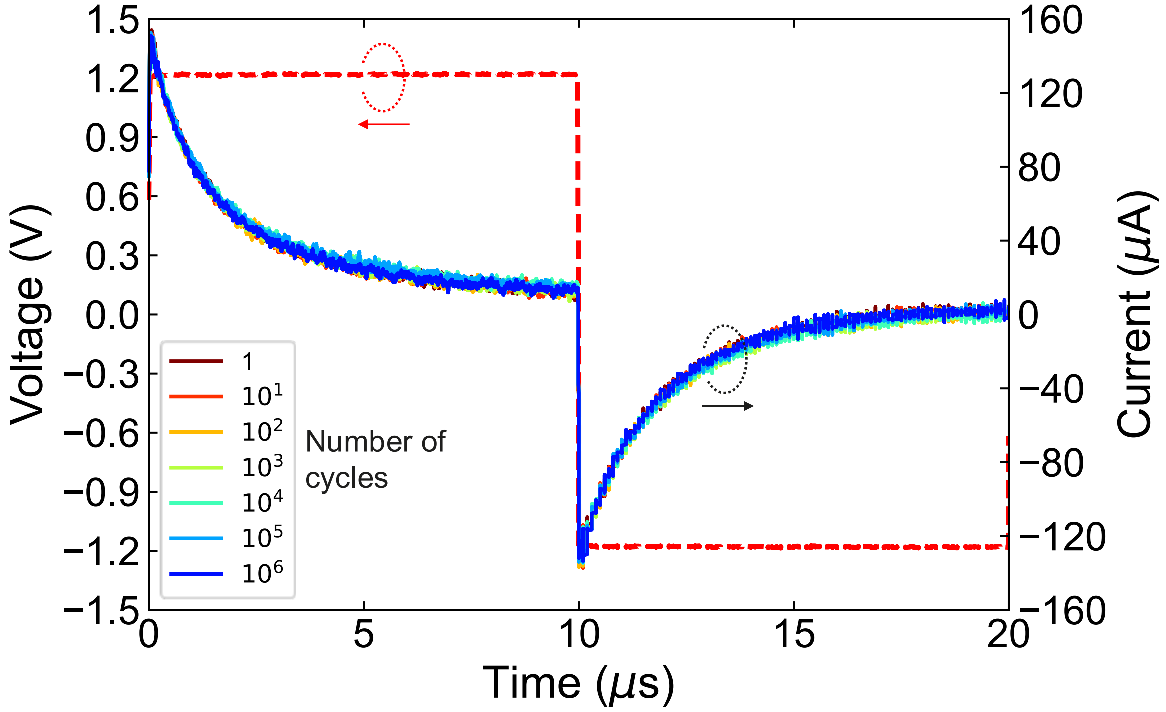

Figure S4. Endurance characteristics of Au/[bmim$^+$][TFSI$^-$]/Au with respect to repetitive voltage pulse application up to 10^6 cycles. In this measurement, voltage pulse of +1.2 V with 10 μs-duration and that of −1.2 V with 10 μs-duration were alternatively applied without any intervals. The device state was checked every single-digit cycle. Red dashed lines indicate applied voltage and solid lines indicate measured current. This measurement can be considered as endurance test repeatedly programming (+1.2 V pulse) and initializing (−1.2 V pulse) the device.

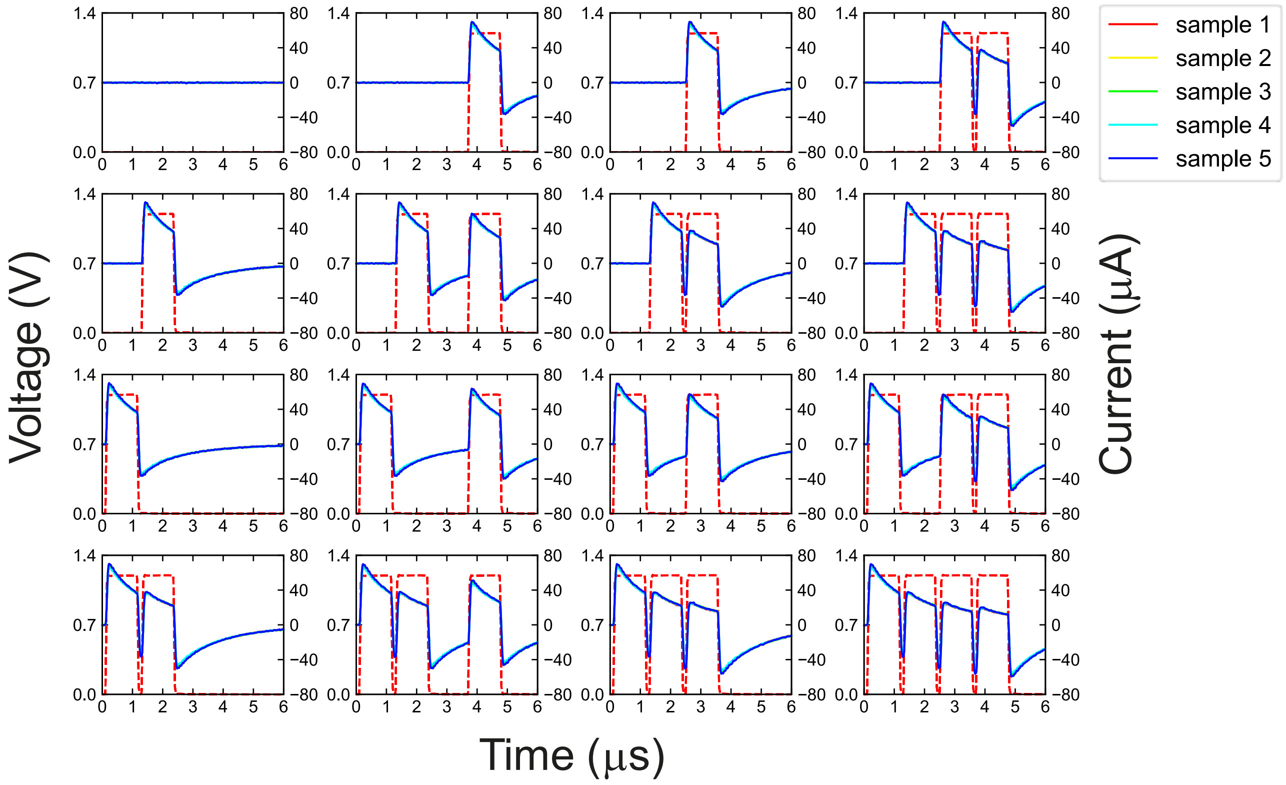

Figure S5. Dynamic current responses to all combinations of 4-bit pulse patterns from "0000" (upper left) to "1111" (bottom right) for five different Au/[bmim$^+$][TFSI$^-$]/Au. Dashed line indicates voltage and solid line indicates current.

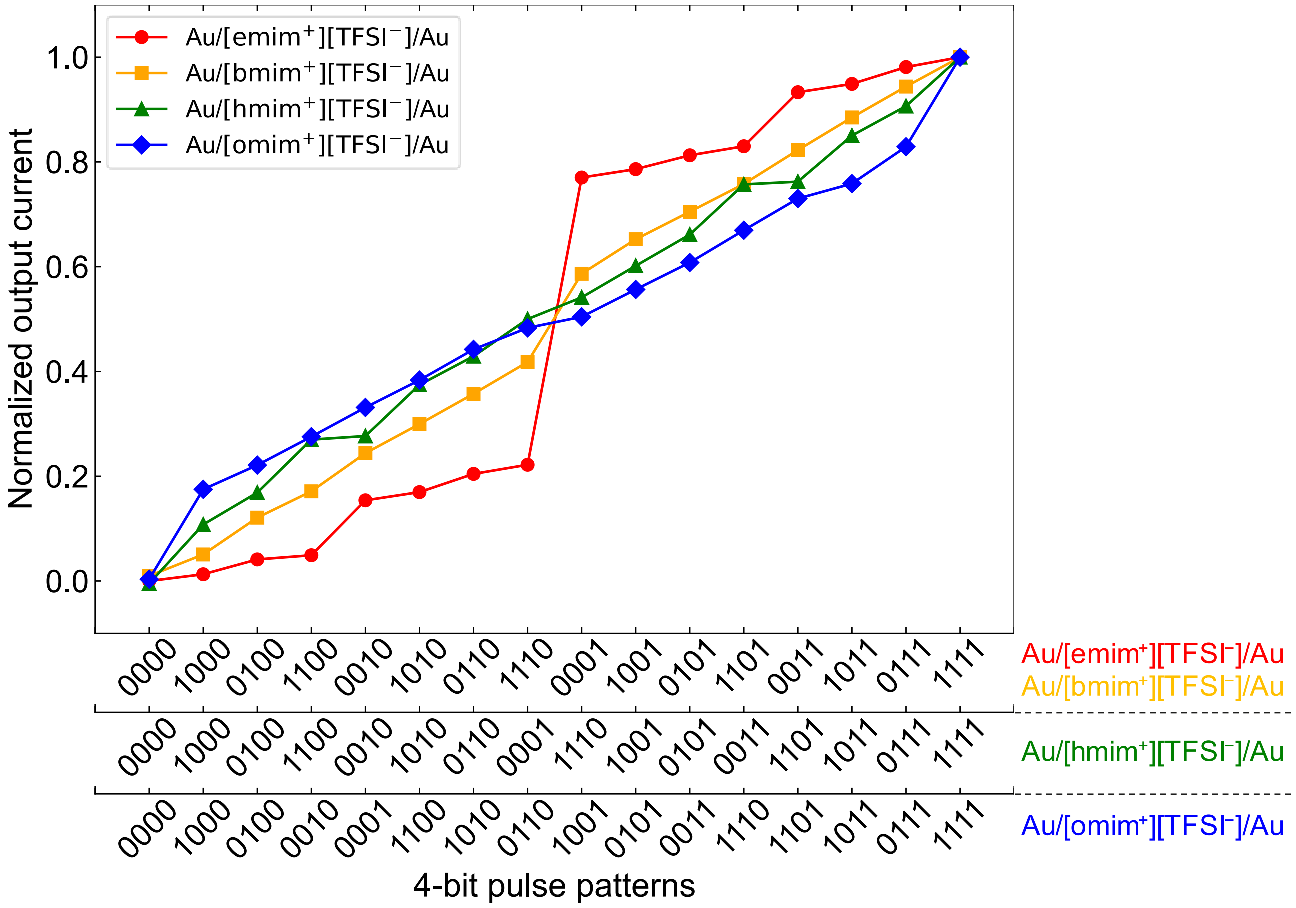

Figure S6. Normalized output current (I_{out}) which was rearranged in ascending order for Au/[emim$^+$][TFSI$^-$]/Au, Au/[bmim$^+$][TFSI$^-$]/Au, Au/[hmim$^+$][TFSI$^-$]/Au, and Au/[omim$^+$][TFSI$^-$]/Au.

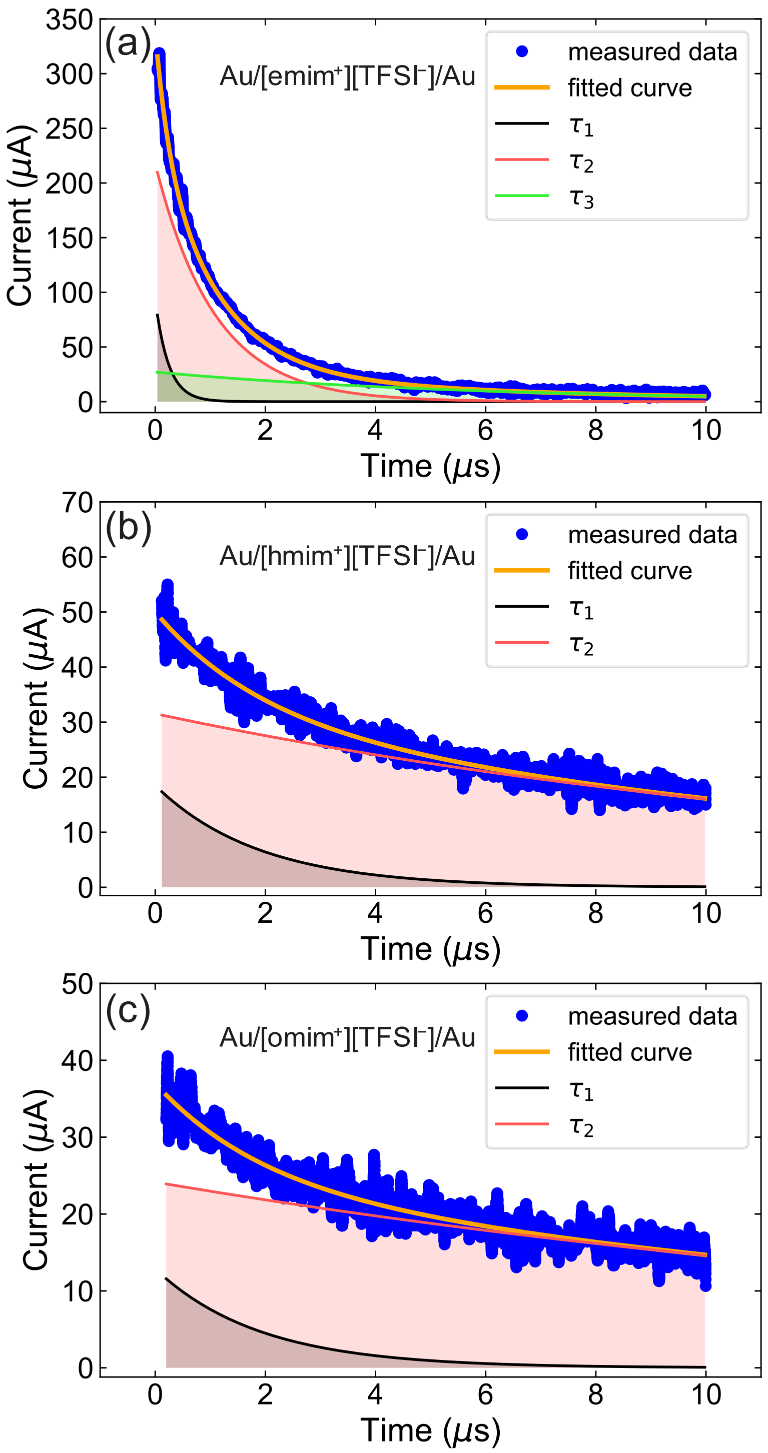

Figure S7. Transient current responses measured with (**a**) Au/[emim$^+$][TFSI$^-$]/Au, (**b**) Au/[hmim$^+$][TFSI$^-$]/Au and (**c**) Au/[omim$^+$][TFSI$^-$]/Au. Their fitted curves simulated based on Eq. (1) are shown by orange solid lines. Debye-type relaxations that constitute the fitted curve are shown by solid line with shade.

Table S1. Parameters extracted by curve fitting shown in Fig. 4b and Fig. S7. Values indicate the average of 7 different devices.

τ_i (s), A_i	τ_1, A_1	τ_2, A_2	τ_3, A_3
Au/[emim$^+$][TFSI$^-$]/Au	2.73×10^{-7}, 1.04×10^{-4}	1.08×10^{-6}, 2.10×10^{-4}	8.43×10^{-6}, 2.04×10^{-5}
Au/[bmim$^+$][TFSI$^-$]/Au	5.48×10^{-7}, 5.40×10^{-5}	2.24×10^{-6}, 8.01×10^{-5}	1.59×10^{-5}, 1.96×10^{-5}
Au/[hmim$^+$][TFSI$^-$]/Au	1.66×10^{-6}, 1.91×10^{-5}	1.29×10^{-5}, 3.32×10^{-5}	-
Au/[omim$^+$][TFSI$^-$]/Au	2.11×10^{-6}, 1.39×10^{-5}	2.18×10^{-5}, 2.36×10^{-5}	-

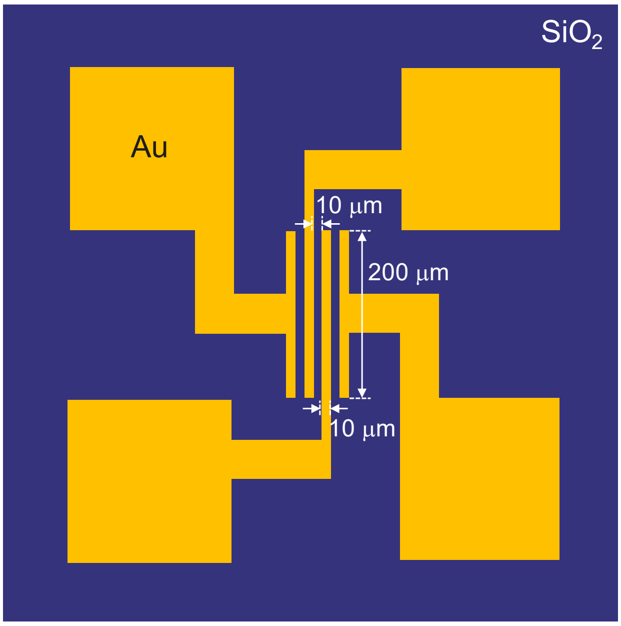

Figure S8. Schematic of Au planar gap electrodes with dimensions of 200 μm-length × 10 μm-width, separated by a 10 μm-gap.
